# The Contribution of MCDM to SUMP: The Case of Spanish Cities during 2006–2021

**DOI:** 10.3390/ijerph19010294

**Published:** 2021-12-28

**Authors:** Salvador Garcia-Ayllon, Eloy Hontoria, Nolberto Munier

**Affiliations:** 1 Department of Civil Engineering, Technical University of Cartagena, Paseo Alfonso XIII, 52, 30203 Cartagena, Spain; 2 Department of Business Economics, Technical University of Cartagena, Calle Real, 3, 30201 Cartagena, Spain; eloy.hontoria@upct.es; 3 INGENIO (UPV), Sir John A. Mc Donald Boulevard, Kingston, ON KTM 1A3, Canada; nolmunier@yahoo.com

**Keywords:** SUMP, urban mobility, city planning, hybrid methodology, multi-criteria decision making (MCDM) methods, SIMUS, weighted sum method (WSM)

## Abstract

Sustainable Urban Mobility Plans (SUMP) are increasingly popular planning tools in cities with environmental issues where numerous actions are usually proposed to reduce pollution from urban transport. However, the diagnosis and implementation of these processes requires broad consensus from all stakeholders and the ability to fit them into urban planning in such a way that it allows the proposals to become realistic actions. In this study, a review of the sustainable urban mobility plans of 47 cities in Spain during the last 15 years has been carried out, analyzing both the diagnosis and proposal of solutions and their subsequent implementation. From the results obtained, a new framework based on a structured hybrid methodology is proposed to aid decision-making for the evaluation of alternatives in the implementation of proposals in SUMP. This hybrid methodology considers experts’ and stakeholders’ opinion and applies two different multi-criteria decision making (MCDM) methods in different phases to present two rankings of best alternatives. From that experience, an analysis based on the MCDM methods called ‘Sequential Interactive Modelling for Urban Systems (SIMUS)’ and weighted sum method (WSM) was applied to a case study of the city of Cartagena, a southeastern middle-size city in Spain. This analytic proposal has been transferred to the practical field in the SUMP of Cartagena, the first instrument of this nature developed after COVID-19 in Spain for a relevant city. The results show how this framework, based on a hybrid methodology, allows the development of complex decision mapping processes using these instruments without obviating the need to generate planning tools that can be transferred from the theoretical framework of urban reality.

## 1. Introduction

According to the United Nations, transport is responsible for 25% of greenhouse gases [1], with cars, buses, vans and other actors in the urban mobility of cities responsible for 70% of these emissions [2]. Urban mobility is one of the phenomena that has undergone a process of great transformation in cities in recent decades [3]. Its guiding parameters and derived impacts are among several of the main Sustainable Development Goals (SDGs) of the 2030 Agenda of the United Nations, having a special impact on factors such as air pollution, the rational use of available resources, efficiency and competitiveness of the labor market and the environmental improvement of public space [4]. Mobility indirectly affects SDG 9, which aims to “Build resilient infrastructure, promote inclusive and sustainable industrialization and foster innovation” and SDG 7 “Affordable and clean energy” where sustainable mobility aims at reducing the use of fossil fuels, and more specifically, SDG 11, which seeks to “Make cities and human settlements inclusive, safe, resilient and sustainable” [5,6].

According to the United Nations [7], planning mobility in the cities of the future is a strategic issue for the sustainable development of the planet. For this reason, its delegations are developing projects to use mobility as a tool to achieve the SDGs. As established by [8], sustainable mobility must be equitable, efficient, green and safe. Several authors [9,10,11] even consider that ensuring mobility in urban areas is essential to achieve at least 7 of the rest of the SDGs: health and well-being (SDG 3), affordable and clean energy (SDG 7), decent work and economic growth (SDG 8), industry, innovation and infrastructure (SDG 9), reduction of inequalities (SDG 10), sustainable cities and communities (SDG 11) and climate action (SDG 13).

A relevant example is the agenda promoted by the Sustainable Transport Division of the Economic Commission for Europe (UNECE, [7]). This comprises of three types of initiatives: analytical, regulatory and capacity building to guide the development of mobility towards the achievement of the SDGs. In its initiatives, the UNECE considers that betting on sustainable mobility as a model of energy transition, the generation of infrastructures for efficient logistics, vehicle regulation, the establishment of investment models in infrastructures, education in road safety and as the axis of urban planning, would have a major impact on up to 13 of the 17 SDGs.

This importance at the international institutional level, however, has not been transmitted sufficiently to the regulatory framework in countries; decision-making in cities being one of the most difficult factors to implement in this matter [8]. The European Union is currently the area in which the most institutional work has been conducted on this matter at the normative field. The regulatory framework for urban mobility began to be developed in 2011 with the publication of the “White Paper on Transport” [12]. In 2013, the so-called “Urban Mobility Package” was approved, which included various initiatives and communications from both the European Parliament and the European Commission [13]. Finally, in 2016 the European strategy for low-emission mobility was published [14]. However, no directive has yet to be approved that specifies urban mobility must be transposed into the national legislation of the different EU countries [8]. Despite this, in the last decade, many cities have developed sustainable urban mobility plans to implement actions and strategies in their urban planning to promote greener mobility and improve urban space by making it more attractive for pedestrians and bicycles. 

One of the best-known cases in this field is that of the city of Paris [15], where the philosophy of the so-called “City of 15 minutes” promotes proximity mobility in urban planning more than the traditional solutions of this type of planning (promotion of public transport, improvement of the cycle lane network, modernization of vehicles, etc.) and has been a success that has been exported internationally. This approach goes beyond the traditional philosophy of sustainable mobility based on the execution of infrastructures and the improvement of the public transport service, proposing a sociological change at the urban level that must be implemented through city planning. Through so-called *chrono-urbanism*, changes are sought to traditional zoning paradigms of cities that segregate uses by promoting long-distance displacements through urban proposals that mix uses in neighborhood structures by covering the main demands of any citizen in a temporary radius, so that their main demands can be carried out walking or cycling. Cities such as Melbourne have adopted similar philosophies with their “20-minute city proposal” [16], while other cities such as Milan, through their *Strade Aperte* plan [17,18], have put forward more restrictive proposals to reduce the space for private vehicle circulation in favor of more space for cycling and pedestrian mobility. 

However, the urban plans to improve mobility are not a recent invention but are being developed through urban planning with different initiatives in some cities that turned out to be pioneers in this matter. This was the case, for example, of the city of Curitiba, which developed the well-known “Integrated transport network” in the 1980s within the framework of the general urban plan of the city, and which later served as a model for many other cities in Latin America such as Medellin, Bogota, Rio de Janeiro and Santiago de Chile [19].

In Spain, the importance of urban mobility has been reflected in the regulatory framework since 2005, and has been supported by generalist decrees, ministerial orders and calls for aid promoting sustainable mobility [20]. This has led to the development of numerous sustainable urban mobility plans (SUMP) in many cities in Spain during the last 20 years, but has not been supported by a specific technical and regulatory framework. This tool has become quite popular in local planning, sometimes more in the effort to obtain subsidies and European funds for cohesion and development, rather than due to the existence of a true political commitment on the part of local administrations [21]. However, the non-existence of a specific regulatory framework on the matter has given rise to a wide technical heterogeneity, with different approaches both at the diagnostic field and at the level of proposing solutions.

One of the best-known proposals in this context in Spain has been the so-called “Madrid Central” plan approved by the Madrid city council in 2018 [22]. This plan restricted access to the urban center area to the most polluting vehicles, and after various political and social controversies is currently paralyzed due to a change of government in the municipal corporation. However, this is not an isolated case, since there are many urban mobility plans in Spain whose implementation has been defective or even non-existent [23,24,25]. This situation has occurred either due to the difficulty in generating the necessary consensus with the stakeholders to establish diagnoses and solutions to the current problems, or due to the lack of will of the municipal administrations when implementing the necessary actions [26].

In this context of policy implementation, the multi-criteria decision making (MCDM) methods approaches are currently a widely used tool in the field of multiparametric analysis for the diagnosis and resolution of complex planning problems. A huge amount of successful cases in their application may be found related to complex scenarios’ assessment [27] in fields such as logistics [28], environmental management [29], construction industry [30], carbon emissions [31] and even sports tourism [32] and medicine management [33]. Therefore, these tools, because of their characteristics, present a high degree of applicability to complex phenomena of urban planning, such as promoting sustainable mobility.

In this study, an analysis of the implementation of sustainable urban mobility plans in Spain in the last 15 years is carried out for 43 cities. Based on the deficiencies detected, a structured framework based on a MCDM hybrid methodology for SUMP implementation is proposed to address most of the common problems observed. This methodology with two phases, considers in the first phase the experts´ opinion and applies the WSM [34] to obtain an initial ranking of best alternatives for urban mobility. To check the robustness of this initial ranking and to correct some problems detected in the analysis performed in 47 sustainable mobility plans, the stakeholders´ priorities are considered in a second phase of the methodology and an additional ranking through the application of SIMUS [35] is presented. In order to check the robustness of the presented methodology and subsequent framework, both rankings are compared to find discrepancies that could make the authors aware of the reliability of the results. 

This methodological proposal has been successfully applied to the case of the sustainable urban mobility plan of the city of Cartagena, the first plan of this category approved after the COVID-19 pandemic in a relevant city in Spain and may be used for other cities that could wish to implement a success sustainable urban mobility plan. 

The rest of the paper is structured as follows. Section 2 develops the methodological proposal and describes the case study: Section 2.1 presents how the analysis was performed for 47 SUMPs carried out in Spain; the proposed methodology and framework with SIMUS and the WSM MCDM methods are explained in Section 2.2; and the case study where the proposed methodology is applied (city of Cartagena) is described in Section 2.3. Section 3 provides the most relevant results that are discussed in Section 4. Finally, the conclusions of this research are available in Section 5. 

## 2. Materials and Methods

The research has been carried out in different phases. First, an analysis has been undertaken to assess the status of the Sustainable Urban Mobility plans carried out in Spain. Based on the information about the weaknesses and strengths detected in these urban planning instruments, it is proposed a methodological approach to improve the development of diagnoses and solution proposals. Finally, this framework has been applied in the case of the city of Cartagena.

### 2.1. Analysis of SUMP Implementation in Spain

For the analysis of the 47 SUMPs evaluated, quantitative and qualitative indicators have been considered to assess the usual profile of the plans. The choice of indicators is aimed at being able to evaluate the statistical correlation between objective parameters of execution and implementation of the plans (quantitative indicators) and parameters of quality and successes of the plans produced (qualitative indicators). The objective is to analyze issues of interest such as, for example, to what extent the existence of a more or less extensive participatory process led to a longer approval time of the SUMP, the relationship between the number of stakeholders involved or the time dedicated to its preparation and the subsequent success of a plan.

The quantitative indicators are the following: 

*L1. Number of stakeholders involved for the diagnosis.* The number of stakeholders referenced in the diagnosis phase of each of the SUMPs has been counted.

*L2. Number of actions proposed in the SUMP.* To avoid statistical biases due to the heterogeneity of the approaches in the different SUMPs, five categories of actions have been established for each plan, limiting the number of actions accounted for each of them to five. The categories are infrastructural actions (e.g., the construction of cycle lanes), actions to enhance the regulatory framework (e.g., parking regulation in low-emission zones), the active promotion of a more sustainable mobility (e.g., the decarbonization of public transport through the purchase of electric vehicles), actions to improve public space (e.g., the development of superblocks or pedestrianization) and actions of an educational nature (e.g., road safety education plans). These actions are not watertight and there may be actions that can be included in both categories, such as the introduction of a tram that could be understood as the building of infrastructure or the implementation of a less polluting mobility system.

*L3. Time necessary for its elaboration (months).* To consider time, the official administrative files or the municipalities’ own websites have been used, establishing indicative dates through news in the newspapers when specific data were not available from official sources.

On the other hand, the qualitative indicators are the following: 

*L4. Amplitude of the participatory process of elaboration*. Four levels of extent have been established: basic level in the case of SUMPs carried out solely as technical consulting work by an external company; intermediate level for SUMPs carried out at the basic level plus workshops including stakeholder participation; high level for those of intermediate level with administrative procedure of regulated public exposure for presentation of allegations by all citizens; and comprehensive level for those with high level plus implementation of mechanisms for subsequent monitoring of actions by the administration.

*L5. Number of compliances, monitoring and verification indicators*. Three assessment levels have been established (low, medium and high) depending on the number of monitoring indicators. Those who did not have indicators or made a scant approach in this matter have been established as low, for those who raised it in a purely theoretical way they have been established as medium, and for those who raised a verifiable comprehensive process or who have carried out subsequent revisions and updates of the plan in the following years were considered high.

*L6. Proven degree of fulfilment of the SUMP*. For the assessment of this indicator, three levels have been established (low, medium, high), taking into account the level of news generated after the approval of the plan. Additionally, we have also considered whether there is any type of monitoring information infrastructure by the municipal administration (low for those who have not generated information after its approval, medium for those who have generated some type of subsequent news flow and high for those who have generated a permanent flow of news through the media or through a specific platform). 

### 2.2. Hybrid MCDM Framework Design

The objective of this methodology is to obtain a reliable ranking of best alternatives in SUMP with which to improve the ease of implementation of SUMPs in cities. To this end, two different MCDM methods will be used: WSM ([36]) and SIMUS ([27]). These two methods will first be briefly described and justified, and then their mixed implementation through a hybrid methodology proposal will be explained.

#### 2.2.1. Weighted Sum Method (WSM)

The WSM is the one most recognizable and simplest of the MCDM methods and its use is sometimes recommended because of the ease of its applicability. WSM is available for a wide spectrum of users including non-technical ones, which is the reason for its choice in this work. This methodological approach is the most common one present in the SUMPs studied when structured proposals for diagnosing problems or strategic selection of alternative solutions are made, since its explanation to city’s stakeholders is quite simple. However, it is important to remember that the weights used must be justified based on objective criteria for the methodology to be robust and reliable.

WSM is applicable when evaluating a set of “m” alternatives by means of a set of “n” criteria. To distinguish the importance of a criterion respective to others, weights are considered and the higher the weight is, the higher the importance of the criterion. The importance of alternative *A_i_* denoted as “*Ai ^WSM −Score^*” when it is evaluated in terms of criterion Cj and when all criteria are considered simultaneously, is defined as follows:

(1) $$A_{i}^{WSM-Score}=\sum_{j=1}^{n}W_{j}A_{ij}\;\;\;for\;i=1\;to\;m\;\forall i$$

where: 

$W_{j}$: relative importance of criterion Cj 

$A_{ij}$: score of alternative *A_i_* when evaluated by means of criterion Cj. 

#### 2.2.2. Sequential Interactive Modelling for Urban Systems (SIMUS) Method

In urban mobility, it is quite common when modelling city planning to deal with many alternatives and a large set of criteria to assess them. In selecting a MCDM method, the chosen one must be suitable to address this situation in a reasonable computing time and without the need of investing in expensive hardware. The software SIMUS (Munier, Canada) approach is based on Lineal Programming and is able to tackle this scenario.

SIMUS is a MCDM method which is immune to rank reversal (RR, [37,38]). RR can be explained with the following example: given a final ranking of best alternatives is B ≽ A ≽ D ≽ C (symbol ‘≽’ means that it is preferred or equal to or precede to; therefore, B is preferred to A, which is preferred to D which is preferred to C). This ranking may change when adding a new project or deleting an existing one, i.e., suppose project A is not considered and the MCDM method is run again. The new ranking should be according to common sense: B ≽ D ≽ C, i.e., conserving the ranking precedence in B, D, C. However, it could be that RR appears and the new ranking is D ≽ C ≽ B, which should not happen.

This approach makes the SIMUS approach more attractive for proposing a hybrid framework in the case of urban mobility planning compared to other MCDM methods such as AHP [36], MOORA [39], ELECTRE [40,41], PROMETHEE [42], SAW [41] or TOPSIS [43], for example. 

The reason why SIMUS does not suffer RR is mainly based on the rigid mathematical structured followed in the Simplex algorithm which is repeatedly used for each new objective [27], improving the value of the functional ‘*Z*’ which is expressed as:

(2) $$Z=\sum_{j=1}^{n}\left(\alpha_{j}\;X_{j}\;\beta_{j}\;Y_{j}\right)\;Maximize/Minimize$$

where:

*X* and *Y* are variables or projects

*α* and *β*: Scores for projects

*j*: Number of projects

The Simplex algorithm works using a tableau that has all the data of the problem ordered in a certain manner. In each iteration, the algorithm selects the best project to enter in a new solution by comparing the contribution of all projects (Cj) in improving the solution from the last iteration (Z_j_), that is, (C_j_ − Z_j_) and obviously chooses that with the greatest difference. Once this selection is made, the Simplex determines the project that must be eliminated from the solution (see [27] for a detailed explanation of parameters). Consequently, if in an existing problem a new project vector that is worse than all the others is added to the system, it will never be considered. By the same token, if a new project is added and is better than another, it will be selected by the same algebraic mechanism. 

#### 2.2.3. Hybrid WSM-SIMUS Framework

For the implementation of this methodology, the first step is to analyze the results obtained from the aforementioned indicators of the 47 cities of Spain. The results of this analysis sheds light on the shortcomings and drawbacks when applying theory to real implementations of a SUMP, giving authors guidelines about suitable alternatives to be implemented and the respective criteria to assess them.

For the assessment of alternatives through a framework, a literature review in this field was performed, searching for methods which better match the objectives of this work. Initial output of the proposed hybrid methodology is a first ranking of alternatives obtained through a committee of experts who scored alternatives by means of selected criteria. These criteria were firstly grouped in five clusters, which were weighted according to their importance. To obtain the ranking, a scored matrix was computed by means of the WSM method.

However, reliability of this first ranking had to be checked due to the subjectivity inherent in some stages of the above processes (mainly when weighting the clusters and at a lower level when scoring the alternatives). Based on former appreciations, a second ranking was needed to check the robustness of the initial one. 

This second ranking firstly considers stakeholders´ preferences and resources (budget, workforce, time for execution, etc.) and later the assessment of a committee of experts. Both contributions (stakeholders and experts) had to be supported by a solid MCDM method and the principal reason why the Sequential Interactive Modelling for Urban Systems (SIMUS) method was chosen. In the case of Cartagena, 6 experts were contacted, and 35 stakeholders participated on a personal level or as representatives of an institution.

**Figure 1:**
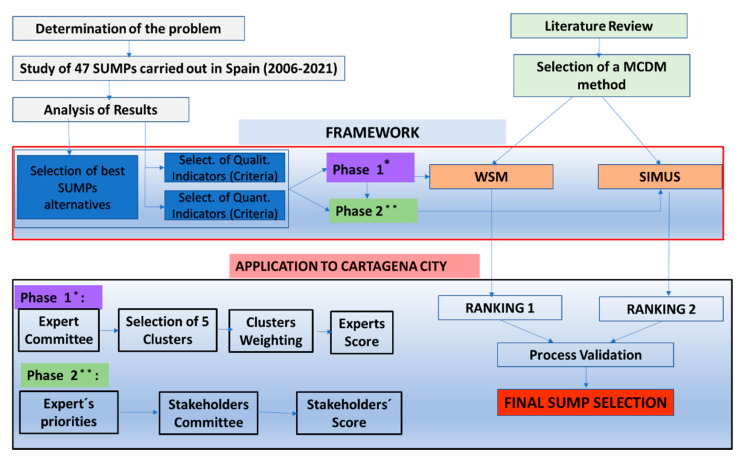
Hybrid WSM–SIMUS methodology proposed for SUMP improvement.

Finally, both rankings were compared searching for high discrepancies that could make the authors aware of shortcomings of the proposed methodology for its application to the city of Cartagena. The process that was followed can be seen in a summarized and schematic way in Figure 1.

The proposed methodology can be summarized in the following steps:

1. Retrospective study of 47 Sustainable Urban Mobility Plans carried out in Spain in the last 15 years.
2. Diagnosis of existing problems in these planning tools and the implementation of strategic guidelines to aid decision-making based on the results obtained.
3. Selection of the best alternatives for a successful SUMP implementation.
4. Selection of a group of criteria to assess former alternatives.
5. Selection of MCDM parameters of methods based on literature review if needed: The Weight Sum Method (WSM) for Phase 1 and the SIMUS method for Phase 2.

**Ranking 1:**

6. Based of literature review, 5 clusters were identified (Atmosphere Quality Improvement, Improvement of Healthy Habits, Enhancement of Competitivity, Public Space Improvement and Social Justice) and strategic goals were grouped as criteria in each one of these 5 clusters.
7. Weighting of the clusters: for the application of the WSM, clusters must be weighted according to the literature review carried out by the technical managers of the Sustainable Urban Mobility Plan.
8. Selection of an expert committee to score alternatives by means of the selected criteria.

Based on the 47 SUMPs studied, the authors have analyzed the main reasons why several of the plans were not finally implemented (lack of consensus between stakeholders, lack of budget, discrepancies between stakeholders and municipal technicians or leading experts of the plan, etc.). To avoid these shortcomings in Phase 2, experts´ opinions, which are the first prioritization, are considered first, deleting and generating priority or conditioned relationships for those alternatives that are understood to pose major problems or require prior procedures. This first selection is based on technical, budgetary or administrative reasons. After this first prioritization, the stakeholder´s selection is conducted with these validated alternatives. In this sense, stakeholders follow a half-guided participatory process avoiding focusing their attention on approaches that lead to unrealizable plans. Both selections (stakeholders and experts) are computed in SIMUS, which provides the second ranking in Phase 2.

**Ranking 2:**

9. Analysis of the experts´ priorities in public workshops or participatory processes.
10. Analysis of available resources (financial, workforce, time, etc.) and preferences based on knowledge of experts and municipal traditional decision makers (political priorities, law limitations, etc.).

Once both rankings have been obtained, a validation process must be carried out contrasting the results obtained for each of the two alternatives proposed to jointly optimize the interests of stakeholders and the limitations imposed by experts or municipal technical managers. 

### 2.3. Application to the Case Study of the City of Cartagena

**Figure 2:**
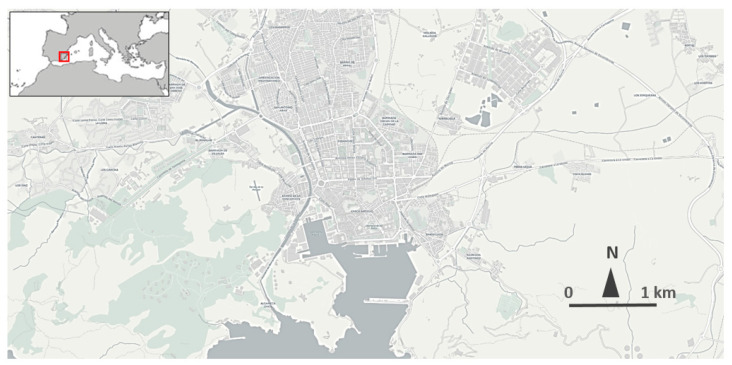
Area of study of Cartagena city. Source: Cartagena city council.

From the analysis of the statistical correlation between these indicators, the implementation of a structured framework for the city of Cartagena (Figure 2), a south-eastern city of Spain, will be assessed.

The city of Cartagena has been chosen because, from a statistical point of view, it is a case approximately located in the median, based on size and population, of the sample of 47 cities selected to carry out the previous analysis of the sustainable urban mobility plans developed in the last 15 years. The city of Cartagena is a medium-sized city (216,000 inhabitants) located in the southeast of Spain. The city has urban and interurban bus lines, commuter, medium and long-distance rail lines, taxi and VTC services (but no presence of multinational technological platforms such as Uber or Cabify) and has an important urban and periurban network of bike lanes. In its urban area, there are about 600,000 trips every day, of which almost 200,000 are made by car. This case does not have the enormous complexity and interaction of multiple phenomena that can occur in cities such as Madrid (3.2 million) or Barcelona (1.6 million), which may mask the impact of other issues not considered in the analysis such as ride hailing, carpooling, the presence of large transport infrastructures or new mobility forms associated with technological development, the collaborative economy or even the underground economy. Nevertheless, it is large enough to accommodate all the typical casuistry in this type of planning instruments, which is not guaranteed in some of the smaller cities analyzed, such as Ciudad Real (77,000) or Teruel (35,000). The sample of 47 selected SUMPs is, in turn, quite statistically representative of Spain as a country, given that there are 50 provinces in the national territory, with most of their capitals included in the analyzed list.

## 3. Results

For the presentation of results, the proposed framework was applied. First, a statistical analysis of the diagnostic indicators of the 43 SUMPs analyzed (See Appendix A) was carried out. In a second phase, the results obtained from the analysis of all these plans have served us to apply the proposed methodological framework in the case study of the Sustainable Urban Mobility Plan of Cartagena.

### 3.1. Analysis of SUMPs in Spain between 2006 and 2021

**Figure 3:**
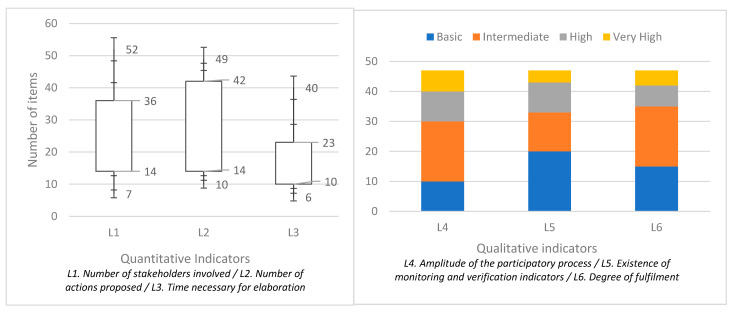
Results obtained for the quantitative and qualitative indicators analyzed in the 43 cities.

First, an analysis has been made of the level of presence from a statistical point of view of the different qualitative and quantitative indicators in the selected sample of 47 plans developed in 43 cities in Spain (four of them made second editions to update the previous plan) during the last 15 years. On the other hand, the level of interaction of the results of qualitative and quantitative statistical indicators has been evaluated, by means of a statistical correlation using a linear decision system by least squares (OLS). The results obtained can be seen in Figure 3 and Table 1 for indicators L1 (number of stakeholders), L2 (number of actions in SUMPs), L3 (number of months for implementation), L4 (amplitude of the participatory process of elaboration), L5 (existence of monitoring and verification indicators) and L6 (proven degree of fulfilment of the SUMP).

If we observe the results obtained, it can be seen that there is a clear statistical correlation between the scarce presence of stakeholders or the absence of a broad participatory process at the SUMP level with the comprehensive proposal of solutions to existing problems. There is also a clear relationship between the absence of follow-up and monitoring mechanisms for these instruments and the failure to achieve their objectives over time. According to what can be observed in the regression coefficients B, the qualitative indicators L4 (amplitude of the participatory process of the SUMP) and L6 (degree of SUMP fulfilment) present the highest level of correlation in general with the quantitative indicators L1 (number of stakeholders involved), L2 (number of actions proposed) and L3 (time necessary for elaboration). On the contrary, the L5 indicator (existence of monitoring and verification indicators) is the one with the weakest regression coefficients, with negative values in some cases (L3 case, close to zero, which rather denotes the absence of correlation). Among the quantitative indicators, the L1 indicator is the one with the most stable behavior, with the L3 being the one with the most heterogeneous values.

In relation to the evaluation of the performance of the model and the relative quality of the statistical model for the given set of data, obtained through the multiple R squared/adjusted R squared values and the Akaike information criterion, respectively, the statement in the previous paragraph is confirmed, with the most robust correlation model being the one that correlates the quantitative indicators with L4, the most robust, and L5, the weakest.

On the other hand, it is interesting to observe how the majority (62%) of the plans analyzed do not fairly contemplate structured methods of diagnosis and selection of alternatives in decision-making aid. Most of them use a rudimentary or qualitative approach type when this issue is addressed (34%). Only a very exceptional minority (4%) raises structured methods based on objective criteria (usually, type AHP or WSM). Finally, it is interesting to find out that, if applied, the implementation of these improvement mechanisms does not necessarily imply the generation of a longer processing time for the approval of the plan. If we delve into the reasons for the lack of success or the difficulty of approval of the mobility plans studied, we find as the most recurrent causes, the absence of consensus between the stakeholders and the municipal technicians in charge of drafting the plans and the lack of available budget to undertake the actions.

### 3.2. Application of the Hybrid Framework for the SUMP of the City of Cartagena

Based on the results obtained in the study of SUMPs in Spain, the previously explained methodology has been proposed to improve the implementation process of these planning instruments in cities. The methodological framework described has been applied to the case study of the city of Cartagena, a city that houses the usual characteristics of the statistical sample selected in the previous study.

As a consequence of the application of the proposed framework in its Phase 1, the following 18 × 22 decision matrix (see Table 2) was obtained after the application of the sum weight method using weighting criteria obtained based on a review of scientific literature. The weighting criteria of the different coefficients have been established based on the following documents: general priorities established in the Transport White Paper “Towards a competitive and efficient transport system in the consumption of resources” [12] published in 2011 by the European Commission and the Sustainable Development Goals 2030 of the United Nations organization (Resolution A/RES/70/1 approved by the General Assembly on 25 September 2015 [6]), and specific technical criteria for strategic mobility planning, such as the practical guide for the preparation and implementation of SUMPs published in 2008 by the IDEA foundation [44] and the conclusions established at the Sustainable Urban Mobility Congress held in Bilbao in 2019 [45].

The weighting criteria of the different alternatives evaluated by the expert committee are described below:

- Improvement of environmental quality (IEQ, 20%):
  ○ Promotion of energy efficiency (EE, quotient × 0.3)
  ○ Improvement of air quality (AQ, quotient × 0.3)
  ○ Promotion of noise reduction (NR, quotient × 0.4)
- Promotion of healthy habits (PHH, 20%):
  ○ Safe and comfortable city for bicycle use (BS, quotient × 0.3)
  ○ Rationalization of the use of the private car (CUR, quotient × 0.2)
  ○ Safe and comfortable city for mobility on foot (PS, quotient × 0.3)
  ○ Promotion of physical exercise (PE, quotient × 0.2)
- Improving competitiveness (IC, 20%):
  ○ Reduction of travel times (TTR, quotient × 0.2)
  ○ Infrastructures for more efficient non-motorized mobility (NEVI, quotient × 0.3)
  ○ Electric vehicle charging infrastructures (EVI, quotient × 0.2)
  ○ Encouragement of bicycle travel (BUI, quotient × 0.3)
- Improvement of public space (IPS, 20%):
  ○ Elimination of architectural barriers (SB, quotient × 0.3)
  ○ Decrease in the occupation of public space by motor vehicles (COR, quotient × 0.2)
  ○ Promotion of comfortable, inclusive and safe mobility (ISM, quotient × 0.3)
  ○ Creation of public space for coexistence (CS, quotient × 0.2)
- Social justice (SJ, 20%):
  ○ Goods accessible to all citizens (HG, quotient × 0.3)
  ○ Reduction of territory fragmentation and barrier effect (EB, quotient × 0.3)
  ○ Better quality of life for inhabitants and passers-by (LQ, quotient × 0.4)

In Table 2, scores assigned by WSM to different actions proposed are presented in columns and the five weighted clusters containing respective criteria are shown in rows.

Each cell contains the score given by the expert committee when assessing an alternative regarding a determined criterion, being the maximum score 9 and minimum 1. For this method, Alternative 3 “Building of an integrated and coherent bicycle lane network” is the one out of 26 which gets the highest score as it can be observed in the last row from Table 2 and is consequently the first alternative.

Derived from Table 2, the first ranking of alternatives is obtained and depicted in Table 3 (only 10 most valued alternatives are shown).

As foreseen in the proposed framework, and aiming to check the robustness of previous Ranking 1, another ranking was obtained after the application of the SIMUS method. For the implementation of this second methodological approach, issues such as the precedence of the actions have been considered for evaluation of alternatives, in order to be able to assess the budgetary needs and technical feasibility of the actions in a combined way when establishing evaluation criteria to the different options. Values obtained with SIMUS algorithm and inputs criteria for scoring the alternatives evaluated can be observed in Table 4.

Table 4 shows the Efficient Result Matrix (ERM) where the 26 alternatives are in columns and the 18 criteria are in rows. The ERM matrix is Pareto Efficient since all scores or results of the different objectives are optimal, that is, they cannot be improved.

Consequently, the final scores for all alternatives are shown in the last row in solid black. According to these scores, Ranking 2 is given and is depicted in Table 5. 

For a better comparison of Ranking 1 and Ranking 2, both are shown vis-a-vis in Table 6 to check differences. Horizontal arrows indicate exact correspondence between the two rankings. Obliquus arrows show the difference in positions between scores of the two rankings.

## 4. Discussion

The analysis carried out for the last 15 years in Spain has been quite revealing about a problem of urban planning in cities that, due to its fairly recent nature, has had little attention paid to it in the current scientific literature [46,47,48,49]. One of the issues found during the bibliographic review of the 47 urban mobility plans analyzed, was the existence of a certain heterogeneity in their approach. 

However, we can distinguish chronologically two distinct groups. In this sense, we can talk about a first-generation urban mobility plan from the first proposals from 2006 to approximately 2013, and a second-generation plan from around 2014 to the present. The date of passage from one period to another does not respond to any specific regulatory or technical milestone, but rather to the fact that by that time almost all the main capital cities of the country had already developed an instrument of this category [26].

Regarding these first-generation plans, it should be noted that it is from the point of view of their approach to fairly heterogeneous proposals, as there was no regulatory pattern beyond certain recommendations made by some institutions [44]. Although from the point of view of its results, we have very unequal cases, at the level of supplementation, it should be noted that in many cases these were proposals rather voluntaristic, and subsequently, there was scarce later implementation. 

Sometimes, the lack of success of this type of plan responds to the absence of a political commitment beyond obtaining the subsidies that the approval of these instruments granted local corporations at that time. However, in other cases, the problems for their implementation stemmed from technical deficiencies in the plans themselves, because of the lack of verification and monitoring tools or due to the absence of a realistic approach in relation to their objectives.

In the case of the so-called second-generation plans, we find a greater homogeneity of approaches, although uneven results remained. In this case, it should be noted that this period includes plans developed for the first time and others from cities that have proceeded to review their sustainable urban mobility plan drawn up during the previous stage to develop a more up-to-date one. 

The greater homogeneity of approaches responds to the existence of a growing technical literature and greater experience of local administrations in this matter, although, there is still no regulatory technical framework available, as it happens at present in other countries [50,51,52,53]. Regarding its results, although the time frame to observe them in this case is much shorter, we found an absence of real commitments by local authorities. In addition, technical or budgetary problems, regarding the difficulty of making realistic approaches and reaching the necessary consensus with stakeholders for the implementation of actions persist. 

If we make a comparison with the performance at an international level, we can find similar phenomena (see for example active travel policies in Italy [54] or regulatory shortcomings for defining SUMPs in Portugal or the Czech Republic [55]) and issues with different problems (large cities casuistry with societies highly aware of sustainable mobility in Europe [56] or problems in Latin America linked to poor urban planning, see [57]). However, the need to implement new analysis methodologies that evaluate the operation and design of SUMPs and help improve their capacity for success continues to be a constant that continues to be raised from different approaches at the international level [58,59,60].

In this context, the framework proposal resulting from this work can be of great help in improving the implementation of new tools, both in the diagnostic and in the solution proposal phase of SUMPs. It is a common situation when dealing with a large set of alternatives to be implemented in a planning tool such as SUMP that some of them may precede others. For example, to implement solar panels to recharge bikes in a bike station, it is first necessary to build bike stations, as well as and perhaps before that stage is the need to build bike lanes in specific areas of a city. 

In the case of decisions based on consensus with stakeholders, this precedence from some alternatives to others is important for several reasons, like the available time to finish the complete project and/or the available budget each year. In the case study of Cartagena, this key factor was performed by computing stakeholders´ precedence by the SIMUS algorithm, the output of which may be observed when comparing alternative 4 ‘Implementation of the use of PMV and electric vehicles’ in both rankings, where a discrepancy of 3 positions is found. As can be seen in Table 6, there have been significant changes in the ranking of preferences for alternatives, the consideration of which, thanks to this hybrid process, avoids several of the problems detected in the study of the 47 SUMPs.

Hired experts and local government officials are well acquainted with these technical, legal or budgetary issues that usually condition the viability of the implementation of these urban planning tools [61]. However, in the current situation, in which social participation has become a backbone of any urban planning proposal, no SUMP can be realistically implemented without the endorsement of numerous stakeholders such as neighborhood associations, users of bicycles and personal mobility vehicles, representatives of affected institutions, merchants, etc. [62].

Therefore, it is necessary to develop more sophisticated and objective tools that can combine these two approaches in a realistic way to avoid the problems detected in the 47 SUMPs analyzed. Regarding the difference obtained in the results between both analysis methodologies, it should be noted that no drastic changes should be expected (which would not make sense), but relevant changes whose introduction optimized using SIMUS and its precedence parameters possibly helps to avoid problems later during the implementation of the SUMP.

In this context, the proposed working methodological framework covers many of the deficiencies detected with the implementation of a hybrid methodology that combines the simplicity of application of an objective WSM methodology based on weighted evaluation indicators and the capability to implement factors of precedence provided by SIMUS. Although the results obtained for the case study of Cartagena do not show a radical difference in the results between the two approaches, they do allow the adjustment of some parameters of prioritization and validation of actions. These improvements may avoid leaving some loose ends, which in many cases may later entail a delay, or even blockage, of the SUMP start-up for budgetary, political or administrative problems. 

The proposed methodological framework has limitations since it implies knowing the internal functioning of the precedence systems of the diagnostic phases and the proposed proposals so that it really allows an effective optimization of the SUMP. This approach has been effective in a medium-sized city such as Cartagena, where the number of variables and their interactions is reasonably manageable. However, it would be interesting for future lines of research to deepen the effectiveness of this methodology by evaluating its ability to optimize processes for proposals for solutions in urban mobility planning instruments in larger and more complex cities such as Madrid or Barcelona.

## 5. Conclusions

The development of Sustainable Urban Mobility Plans in numerous cities in Spain during the last 15 years without the existence of a specific regulatory framework has given rise to a varied catalogue of actions with various problems. In this study, 47 plans of this nature developed in different Spanish cities have been analyzed, observing how many of these planning instruments have had difficulties both in diagnosing problems and in implementing solutions. By means of a statistical analysis, it has been contrasted how there is a clear correlation between the implementation of these instruments with rudimentary participatory processes or the scarcity of indicators for subsequent monitoring with the failure of these strategies to improve urban mobility in cities.

Based on this diagnosis, a structured hybrid MCDM framework based on WSM and SIMUS methods has been proposed. The results obtained with the application of this methodology for the implementation of the SUMP of the city of Cartagena, show how the implementation of analytic mechanisms in the SUMPs of middle-sized cities such as Cartagena can facilitate the achievement of their objectives.

## Figures and Tables

**Table 1:** Regression analysis using OLS of the existing statistical correlation between quantitative and qualitative indicators.

Indicators	Amplitude of the Participatory Process (L4)				Compliance Indicators (L5)			
	B	Std. Error	*t*	Sign.	B	Std. Error	*t*	Sign.
L1	0.245	0.09	3.384	0.000 *	0.108	0.08	1.992	0.000 *
L2	0.196	0.06	2.767	0.000 *	0.054	0.07	1.682	0.000 *
L3	0.097	0.07	1.810	0.000 *	−0.032	0.09	−3.023	0.000 *
Akaike’s information criterion (AIC): 24,304.5					AIC: 21,009.1			
Multiple R-squared: 0.22					Multiple R-squared: 0.18			
Adjusted R-squared: 0.22					Adjusted R-squared: 0.17			
F-statistic: 121.09 Prob (>F) (3,3) degrees of freedom: 0					F-statistic: 107.34 Prob (>F) (3,3) DF: 0			
**Indicators**	**Proven degree of fulfilment of the SUMP (L6)**							
	**B**	**Std. error**	***T***	**Sign.**				
L1	0.219	0.04	2.476	0.000 *				
L2	0.285	0.06	1.232	0.000 *				
L3	0.270	0.05	1.899	0.000 *				
Akaike´s information criterion (AIC): 22,736.9								
Multiple R-squared: 0.22								
Adjusted R-squared: 0.21								
F-statistic: 143.64 Prob (>F) (3,3) degrees of freedom: 0								

* Significant at 0.01 level.

**Table 2:** Decision Matrix Assessment by Expert Committee with Alternatives in columns and Criteria in files.

		Promote Pedestrian Isplacement	Commuter Services	Bike Lanes	Electric MPV	Foster Bike Use	Car Parking	Taxi Fostering	Bus Lines Optimization	Smart Paths	Green Paths	City Center and Suburbs Connections	Superblocks	Road Safety	Pedestrian Center	Intercity Road Safety	Parking Management	Last Mile Logistics	Work Place Transport	IT Transport Manag.	School Paths	Intercity Public Transport	Collab. Public Transport	Traffic Issues Management	20/30 Zones	Intermodality	Cabo Palos Railway
**IEQ**	EE	1	1	1	9	1	1	1	7	1	1	1	1	1	1	1	1	1	1	7	1	7	7	1	1	7	1
	AQ	9	3	9	8	9	1	1	3	9	9	1	5	1	9	1	1	1	1	1	7	3	3	7	7	3	1
	NR	9	3	9	9	9	5	1	1	9	9	1	5	5	9	5	3	3	3	1	7	1	1	5	5	3	1
**PHH**	BS	9	1	9	7	9	1	1	1	9	9	1	7	7	9	7	1	1	7	1	9	1	1	3	7	3	1
	PS	9	1	3	3	3	1	1	1	9	9	1	7	5	9	5	1	1	7	1	9	1	1	2	7	1	1
	CUR	9	9	9	9	9	9	3	7	7	9	9	7	1	9	1	3	3	3	1	7	7	7	7	7	8	7
	PE	9	1	9	1	9	1	1	1	9	9	1	5	1	9	1	1	1	7	1	8	1	1	1	1	1	1
**IC**	TTR	1	7	9	9	1	1	5	9	9	9	9	1	7	1	7	7	7	7	8	7	7	7	8	1	6	1
	NEVI	9	3	9	9	9	1	1	1	9	9	1	7	5	9	5	1	1	6	1	9	1	1	3	1	3	1
	EVI	1	1	1	1	1	1	1	1	1	1	1	1	1	1	1	1	1	1	7	1	1	1	1	1	3	1
	BUI	3	1	9	3	9	1	1	1	8	8	1	7	5	3	5	1	1	6	7	9	1	1	3	7	7	1
**IPS**	SB	9	7	9	1	1	3	1	1	3	3	1	9	9	9	9	1	1	5	5	7	7	7	3	9	9	1
	COR	9	9	9	9	9	5	1	8	9	9	8	8	1	9	1	8	8	7	1	9	8	8	7	8	7	7
	ISM	9	7	9	9	9	6	7	9	9	9	9	9	9	9	9	4	4	8	7	9	9	9	6	9	9	4
	CS	9	1	9	1	9	9	7	1	9	9	1	9	9	9	9	7	7	7	1	9	1	1	1	8	8	1
**SJ**	HG	9	7	9	1	1	3	1	1	3	3	1	9	9	9	9	1	1	5	5	7	7	7	3	9	9	1
	EB	9	7	9	1	1	3	1	1	3	3	1	9	9	9	9	1	1	5	5	7	7	7	3	9	9	1
	LQ	9	3	9	9	9	5	1	3	9	9	1	5	5	9	5	3	3	3	1	7	3	7	7	7	3	1
	**SCORE**	**7.48**	**4.04**	**7.80**	**5.78**	**6.04**	**3.26**	**1.88**	**3.08**	**6.80**	**6.92**	**2.56**	**6.12**	**5.04**	**7.48**	**5.04**	**2.38**	**2.38**	**4.64**	**3.32**	**7.00**	**4.08**	**4.40**	**4.06**	**5.84**	**5.46**	**1.78**

**IEQ:** Improvement of environmental quality (**EE**: Energy Efficiency; **AQ**: Air Quality; **NR**: Noise Reduction)/**PHH**: Promotion of healthy habits (**BS**: Bike Safety; **PS**: Pedestrian Safety; **CUR**: Car Usage Reduction; **PE**: Physic Exercise)/**IC**: Improving competitiveness (**TTR**: Travel Time Reduction; **NEVI**: Non-Engined Vehicles Infrastructure; **EVI**: Electric Vehicle Infrastructure; **BUI**: Bike Usage Increasement)/**IPS**: Improvement of public space (**SB**: Stop Barriers; **COR**: Car Occupation Reduction; **ISM**: Inclusive/Safety Mob.; **CS**: Coexistence Space)/**SJ**: Social Justice (**HG**: Handy Goods; **EB**: Electric Barriers; **LQ**: Life Quality).

**Table 3:** Ranking 1 after Phase 1 of the methodology and application of the WSM.

Ranking Position	Alternative Number	Alternative	Score
1	3	Building of an integrated and coherent bicycle lane network	7.8
2	1	Promotion of pedestrian movements	7.48
3	14	Pedestrianization of the Historic Center	7.48
4	20	Generation of safe school itineraries	7
5	10	Greenway Connection	6.92
6	9	Start-up of smart trails	6.80
7	12	Traffic calming through superblocks	6.12
8	5	Recovery from bicycle use	6.04
9	24	Deploy zones 30 and 20 min	5.84
10	4	Implementation of the use of PMV and electric vehicles	5.78

**Table 4:** SIMUS Efficient Result Matrix (ERM) and ranking of alternatives for Cartagena´s City SUMP.

	Promote Pedestrian Displacement	Commuter Services	Bike Lanes	Electric MPV	Foster Bike Use	Car Parking	Taxi Fostering	Bus Lines Optimization	Smart Paths	Green Paths	City Center and Suburbs Connections	Superblocks	Road Safety	Center Pedestrianization.	Intercity Road Safety	Parking Management	Last Mile Logistics	Work Place Transport	IT Transport Manag.	School Paths	Intercity Public Transport	Collab. Public Transport	Traffic Management	20/30 Zones	Intermodality	Cabo Palos Railway
EE		0.06		0.06	0.06	0.06		0.06	0.06	0.06		0.06	0.06		0.06			0.06	0.06		0.06	0.06	0.06	0.06	0.06	
AQ	0.33		0.33											0.33												
NR	0.33		0.33											0.33												
BS	0.25		0.25											0.25						0.25						
PS									0.50	0.50																
CUR	0.33		0.33											0.33												
PE	0.33		0.33											0.33												
TTR									0.50	0.50																
NEVI	0.25		0.25											0.25						0.25						
EVI		0.07		0.07	0.07				0.07	0.07		0.07	0.07		0.07			0.07	0.07		0.07	0.07	0.07	0.07	0.07	
BUI			1.00																							
SB	0.33		0.33											0.33												
COR	0.25		0.25											0.25						0.25						
ISM		0.03		0.09	0.09				0.09	0.09		0.09	0.09		0.09			0.03	0.03		0.03	0.03	0.03	0.09	0.09	
CS	0.25		0.25											0.25						0.25						
HG	0.33		0.33											0.33												
EB	0.33		0.33											0.33												
LQ	0.33		0.33											0.33												
**SOC**		0.06		0.06	0.06	0.06		0.06	0.06	0.06		0.06	0.06		0.06			0.06	0.06		0.06	0.06	0.06	0.06	0.06	
**PF**	3.67	0.16	4.67	0.22	0.22	0.06	0.00	0.06	1.22	1.22	0.00	0.22	0.22	3.67	0.22	0.00	0.00	0.16	0.16	1.00	0.16	0.16	0.16	0.22	0.22	0.00
**NPF**	12	3	13	3	3	1	0	1	5	5	0	3	3	12	3	0	0	3	3	4	3	3	3	3	3	0
**RESULT**	**0.67**	**0.17**	**0.72**	**0.17**	**0.17**	**0.06**	**0.00**	**0.06**	**0.28**	**0.28**	**0.00**	**0.17**	**0.17**	**0.67**	**0.17**	**0.00**	**0.00**	**0.17**	**0.17**	**0.22**	**0.17**	**0.17**	**0.17**	**0.17**	**0.17**	**0.00**

**EE**: Energy Efficiency; **AQ**: Air Quality; **NR**: Noise Reduction; **BS**: **Bike** Safety; **PS**: Pedestrian Safety; **CUR**: Car Usage Reduction; **PE**: Physic Exercise; **TTR**: Travel Time Reduction; **NEVI**: Non-Engined Vehicles Infrastructure; **EVI**: Electric Vehicle Infrastructure; **BUI**: Bike Usage Increasement; **SB**: Stop Barriers; **COR**: Car Occupation Reduction; **ISM**: Inclusive/Safety Mob.; **CS**: Coexistence Space; **HG**: Handy Goods; **EB**: Electric Barriers; **LQ**: Life Quality. **SIMUS OUTPUTS** (IN GREY COLOUR): **SOC** = SUM OF COLUMNS; **PF** = PARTICIPATION FACTOR; **NPF** = NORMALIZED PARTICIPATION FACTOR. **RANKING: Alt.3—Alt.1—Alt.14—Alt.9—Alt.10—Alt.20—Alt.4—Alt.5—Alt.12—Alt.13—Alt.15—Alt.24—Alt.25—Alt.2—Alt.18—Alt.19—Alt.21—Alt.22—Alt.23—Alt.6—Alt.8—Alt.7—Alt.11—Alt.16—Alt.17—Alt.26.**

**Table 5:** Ranking 2 after Phase 2 of the methodology and the application of SIMUS method (only 10 most valued alternatives are shown).

Ranking Position	Alternative Number	Alternative	Score
1	3	Building of an integrated and coherent bicycle lane network	0.72
2	1	Promotion of pedestrian movements	0.67
3	14	Pedestrianization of the Historic Center	0.67
4	9	Start-up of smart trails	0.28
5	10	Greenway Connection	0.28
6	20	Generation of safe school itineraries	0.22
7	4	Implementation of the use of VMP and electric vehicles	0.17
8	5	Recovery from bicycle use	0.17
9	12	Traffic calming through superblocks	0.17
10	13	Road safety improvements	0.17

**Table 6:** Comparison of Ranking 1 and Ranking 2 to validate the proposed methodology.

Alternatives	Ranking 1 Best Alternatives	Ranking 2 Best Alternatives	Scores WSM (Ranking 1)	Scores SIMUS (Ranking 2)
Building a bicycle lane network	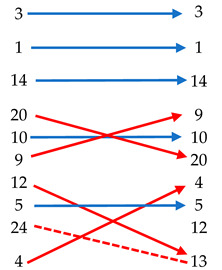		7.8	0.72
Promotion of pedestrian movements			7.48	0.67
Pedestrianization of the Historic Center			7.48	0.67
Generation of safe school itineraries			7	0.28
Greenway Connection			6.92	0.28
Start-up of smart trails			6.80	0.22
Traffic calming through superblocks			6.12	0.17
Recovery from bicycle use			6.04	0.17
Deploy zones 30 and 20 min			5.84	0.17
Implementation of the use of PMV and electric vehicles			5.78	0.17

## Appendix A

The cities where SUMPs have been analyzed for the study are as follows:

**Table A1:** List of cities in Spain with SUMP analyzed.

City	Start Date	Approval Date	Source
Madrid	March 2013	December 2014	madrid.es
Barcelona	September 2012 December 2020	March 2015 In process	barcelona.cat barcelona.cat
Vitoria-Gasteiz	2005 September 2019	October 2007 In process	vitoria-gasteiz.org vitoria-gasteiz.org
Sevilla	2019	May 2021	sevilla.org
Málaga	2015	July 2020	movilidad.malaga.eu
Valencia	2011	December 2013	www.upv.es
Murcia	2009	May 2013	www.murcia.es
Alicante	2011	December 2013	www.alicante.es
Bilbao	2007 2016	2011 May 2018	pmus.bilbao.eus pmus.bilbao.eus
Vigo	2011 2021	June 2014 In process	hoxe.vigo.org hoxe.vigo.org
Burgos	2005 November 2018	2006 In process	aytoburgos.es aytoburgos.es
Terrasa	No data 2014	2002 May 2016	terrassa.cat terrassa.cat
Santander	2008	February 2010	santander.es
Albacete	January 2007	July 2010	albacete.es
Badalona	2009	June 2015	badalona.cat
Elche	2013	2015	elche.es
Castellon	2007	2009	castello.es
Ponferrada	November 2007	June 2014	ponferrada.org
Leganes	2008	July 2010	leganes.org
Fuenlabrada	2006 2016	September 2008 November 2019	ayto-fuenlabrada.es ayto-fuenlabrada.es
San Vicente de Raspeig	2006 2021	2008 In process	raspeig.es raspeig.es
Torrejón de Ardoz	2019	May 2021	ayto-torrejon.es
Reus	2010	March 2012	reus.cat
S. Fernando de Henares	2007	2009	ayto-sanfernando.com
Palma de Mallorca	2012	October 2014	mobipalma.mobi
Ourense	2011	May 2012	ourense.gal
San Sebastian	2006	September 2008	donostiafutura.com
Tarragona	2010	September 2012	tarragona.cat
Gerona	October 2012	December 2014	web.girona.cat
Lleida	2008	November 2011	mobilitat.paeria.cat
Zaragoza	2006 October 2016	2008 March 2019	zaragoza.es zaragoza.es
Valladolid	2005 2019	November 2007 April 2021	pimussva.es pimussva.es
Córdoba	April 2011	October 2013	pmus.cordoba.es
Jaen	March 2021	In process	pmusjaen.com
Granada	2010	February 2013	movilidadgranada.com
Ciudad Real	2010	March 2012	ciudadreal.es
Cádiz	2012	June 2013	institucional.cadiz.es
Salamanca	2011	Julio 2013	ingenieriacivil.aytosalamanca.es
Logroño	2011	November 2013	logroño.es
Teruel	2010	Junio 2012	urbanteruel.es
La Coruña	2011	December 2013	coruna.gal
Pontevedra	2016	April 2020	aestrada.gal
Lorca	2016	May 2017	movilidad.lorca.es
Cartagena	October 2020	September 2021	cartagena.es

Technical note: the number of SUMPs analyzed to prepare the statistical analysis with indicators is 47 SUMPs from 43 cities in Spain. This is because there are 4 cities that have two versions of their SUMP on this question, it must also be specified that in the list in the appendix there are 10 cities in this situation; however, 6 of them have recently finished their second version of their SUMP or they still have it in process, so for the purposes of statistical analysis, they have not been able to be computed since they do not have enough background to analyze the compliance indicator L6. City 44 is Cartagena, the city of the case study, which, like the one mentioned, approved its SUMP recently, so it has not been computed in the statistical analysis of Phase 1.
